# *Orientia tsutsugamushi* in Human Scrub Typhus Eschars Shows Tropism for Dendritic Cells and Monocytes Rather than Endothelium

**DOI:** 10.1371/journal.pntd.0001466

**Published:** 2012-01-10

**Authors:** Daniel H. Paris, Rattanaphone Phetsouvanh, Ampai Tanganuchitcharnchai, Margaret Jones, Kemajittra Jenjaroen, Manivanh Vongsouvath, David P. J. Ferguson, Stuart D. Blacksell, Paul N. Newton, Nicholas P. J. Day, Gareth D. H. Turner

**Affiliations:** 1 Mahidol-Oxford Tropical Medicine Research Programme, Faculty of Tropical Medicine, Mahidol University, Bangkok, Thailand; 2 Center for Tropical Medicine, Nuffield Department of Clinical Medicine, Churchill Hospital, Headington, Oxford, United Kingdom; 3 Wellcome Trust-Mahosot Hospital-Oxford Tropical Medicine Research Collaboration, Mahosot Hospital, Vientiane, Lao People's Democratic Republic; 4 Nuffield Department of Clinical Laboratory Sciences, Oxford University, Oxford, United Kingdom; University of Texas Medical Branch, United States of America

## Abstract

Scrub typhus is a common and underdiagnosed cause of febrile illness in Southeast Asia, caused by infection with *Orientia tsutsugamushi*. Inoculation of the organism at a cutaneous mite bite site commonly results in formation of a localized pathological skin reaction termed an eschar. The site of development of the obligate intracellular bacteria within the eschar and the mechanisms of dissemination to cause systemic infection are unclear. Previous postmortem and *in vitro* reports demonstrated infection of endothelial cells, but recent pathophysiological investigations of typhus patients using surrogate markers of endothelial cell and leucocyte activation indicated a more prevalent host leucocyte than endothelial cell response *in vivo*. We therefore examined eschar skin biopsies from patients with scrub typhus to determine and characterize the phenotypes of host cells *in vivo* with intracellular infection by *O. tsutsugamushi*, using histology, immunohistochemistry, double immunofluorescence confocal laser scanning microscopy and electron microscopy. Immunophenotyping of host leucocytes infected with *O. tsutsugamushi* showed a tropism for host monocytes and dendritic cells, which were spatially related to different histological zones of the eschar. Infected leucocyte subsets were characterized by expression of HLADR+, with an “inflammatory” monocyte phenotype of CD14/LSP-1/CD68 positive or dendritic cell phenotype of CD1a/DCSIGN/S100/FXIIIa and CD163 positive staining, or occasional CD3 positive T-cells. Endothelial cell infection was rare, and histology did not indicate a widespread inflammatory vasculitis as the cause of the eschar. Infection of dendritic cells and activated inflammatory monocytes offers a potential route for dissemination of *O. tsutsugamushi* from the initial eschar site. This newly described cellular tropism for *O. tsutsugamushi* may influence its interaction with local host immune responses.

## Author Summary

Scrub typhus is an infectious disease common in Southeast Asia, caused by the intracellular bacteria *Orientia tsutsugamushi*. These microorgansims infect humans through chigger mite bites, after which *O. tsutsugamushi* invades cells in the dermis, causing an inflammatory lesion called an eschar. Other rickettsia causing different forms of typhus preferentially invade and grow within endothelial cells lining blood vessels, before spreading systemically. In scrub typhus, unambigous endothelial infection with the bacterium *O. tsutsugamushi* has been described in autopsy samples. In this study, we examined *ex vivo* skin biopsies from eschars of scrub typhus patients admitting to hospital and defined the phenotypes of host cells that *O. tsutsugamushi* invades using specialized immunofluorescence microscopy. In this early phase of disease, we found that endothelial cell infection was rare, but infection of host monocytes and specialist antigen presenting cells, such as dendritic cells, was common. This represents a novel cellular tropism for *O. tsutsugamushi*, which provides a potential route for bacterial spread to the rest of the body and may affect its interaction with the host immune response.

## Introduction

Scrub typhus is a common and neglected disease caused by *Orientia tsutsugamushi* in Southeast Asia, accounting for up to 28% of non-malarial fevers in a prospective fever study in Lao PDR [1], and causing an estimated 1 million clinical cases worldwide every year [2]. Recognition of the disease is difficult, due to its overlapping clinical spectrum with other common causes of fever in this population [3] and due to limitations of current diagnostic methods [4]. As the early diagnosis of typhus disease is key to directing appropriate therapy, understanding how the organism develops, disseminates within the host, and interacts with the cells of the host immune response is important.

The proportion of acute primary infections with development of an eschar at the site of the chigger bite can vary widely with geographical areas, exposure of the population and level of endemicity of scrub typhus. In eastern Taiwan the percentage of eschars observed has been reported at 23% [5], in Japan a study observed 97% [6], and in Thai children from a highly endemic area this was 7% [7]. Accompanying lymphadenopathy is very common in patients with scrub typhus [1], [8], which was also observed in volunteers infected with laboratory-reared chiggers [9]. Fever, rash and non-specific symptoms are common, but the clinical course can be complicated by meningo-encephalitis, a disseminated intravascular coagulation (DIC)-like syndrome or severe pneumonitis, which may culminate in acute respiratory distress syndrome (ARDS) and death [10]–[12].

Definitive evidience of endothelial tropism of *O. tsutsugamushi* requires immunophenotyping and ultrastructural co-localisation, which is available in one report of human post-mortem autopsies [13]. Further data in the literature refers to histopathological reports from patient eschar biopsies and animal studies. Histopathological studies of eschars in humans [14]–[18] and cynomolgus monkeys [19] have described perivascular collections of mononuclear cells, including lymphocytes, plasma cells and macrophages. In recent studies, immunophenotyping revealed a dominance of CD3+ T-cells and CD68+ monocyte/macrophages within infiltrates [15] and an association of *O. tsutsugamushi* with the epithelial lining covering the surface of the sweat ducts and glands [14]. Eschars can have high bacterial loads and have been shown to be useful specimens for both PCR-based and immunohistochemical diagnosis [14], [20].

Parallel studies of human *ex vivo* eschar biopsies in the spotted fever group (SFG) rickettsioses show evidence for infection of endothelium and surrounding leucocytes, with a predominance of neutrophils in the infiltrates in cases of *R. africae* [16], *R. rickettsii* [17] and *R. conorii* [18] although lacking ultrastructural demonstration. In summary, the literature provides limited evidence for the *in vivo* cellular tropism of *O. tsutsugamushi* in humans, due to a lack of co-localization studies allowing phenotypic characterization of the infected leucocyte subsets.

The eschar offers an accessible model for studying *O. tsutsugamushi* interactions during early infection of the human host, including colocalization studies for phenotyping of infected cells to elucidate the immune mechanisms involved in control or dissemination of *O. tsutsugamushi* from the local inoculation site. The study was designed to examine the cellular tropism of *O. tsutsugamushi* in eschars of patients with scrub typhus, characterize host leucocyte responses and determine the role of endothelial infection by *O.tsutsugamushi* in early disease.

## Materials and Methods

### Ethical statement

The study was approved by the National Ethics Committee For Health Research, Ministry of Public Health, Lao PDR, and the Oxford Tropical Research Ethics Committee, UK. Patients were enrolled at Mahosot hospital, Vientiane, Lao PDR after the written consent (translated into Lao) of each patient was obtained by local clinicians. An additional separate written consent (translated into Lao) of each patient was obtained before performing the skin biopsy.

### Patients

The clinical diagnosis of scrub typhus was confirmed with a positive scrub typhus immunochromatographic rapid diagnostic test (RDT; Panbio, Australia) and 4.5 mL of whole blood was taken into EDTA and serum separately for rickettsial culture, PCR and serology testing.

Only patients who had a visible eschar were asked to participate. During the course of the study, 1467 patients with a clinical diagnosis of scrub typhus were tested using serology, culture and/or PCR diagnosis. Of these, 188/1467 (12.8%) were confirmed to have scrub typhus by RDT and of these patients 57/188 (30.3%) had visible eschars. Only 14/57 patients (24.5%) had eschars on a part of the body appropriate for a biopsy (ie not the genitals or exposed areas) and gave consent for biopsy. No patients declined consent. 131/188 (69.7%) of confirmed scrub typhus patients lacked an eschar and were therefore not approached for inclusion in this study.

### Eschar biopsies

Skin biopsies were performed with disposable 3 mm circular punch biopsies (Stiefel Laboratories Inc., Offenbach, Germany) to sample a part of the central necrosis with perifocal inflamed skin, after local anesthesia with 1% lidocaine. Each patient had one biopsy, which was fixed using one of three different methods. The biopsies were either i) fixed in 10% Formalin and processed later into paraffin blocks (n = 7), ii) or stored in 2.5% glutaraldehyde for electron microscopy (n = 4), iii) or frozen in OCT for immunohistochemistry (n = 3, total cases = 14). The cases used in this study were the formalin fixed and EM cases (n = 11), shown in Table 1.

**Table 1:** Details of antibodies used in this study.

Antibody Target	Cellular Specificity	Clone Name	Source	Dilution
CD1a	Dendritic Cells	NA1/34	DAKO	1∶100
CD3	Pan-T cell	F7.2.38	DAKO	1∶ 50
CD4	T-cell (T_H_)	NCL-CD4-368	Novocastra	1∶80
CD8	T-cell (T_C_)	C8/144	LRF	1∶10
CD11c	Neutrophils/Monocytes	Protein 150,95	DAKO	1∶100
CD14	Macrophages	NCL-L-CD14-223	Novocastra	1∶75
CD15	Neutrophils/Granulocytes	By87	LRF	Undiluted
CD20	B-cells	L26	LRF	1∶10
CD31	Endothelial cells	JC70A	LRF	1∶5
CD34	Endothelial cells	Qbend10	Abcam	1∶100
Von Willebrand Factor (FVIIIRA)	Endothelial cells	A0082	DAKO	1∶80
CD68	Monocyte/Macrophages	PGM-1	LRF	Undiluted
CD163	Activated macrophages	Ber-MAC3	DAKO	1∶100
CD209/299	Dendritic cells	DC28	R&D systems	1∶200
S100	Dendritic cells	Polyclonal rabbit anti-human S100A4 serum	DAKO	1∶400
Factor XIIIa	Dendritic cells	E980.1	Novacastra	1∶50
HLADR	CR3/43	CR3/43	LRF	1∶2
BCL11a	B cells		LRF	1∶10
LSP1	B cells, monocytes, granulocytes, some activated T cells	TPD153	LRF	Undiluted
Neutrophil Elastase	Neutrophils	NP57	LRF	Undiluted
Lysozyme	Macrophages/Histiocytes	pRb	DAKO	1∶300
Cytokeratin	Pan cytokeratin cocktail to epithelial cells	Cocktail of LP34 (ab49289) and MNF116 (ab756)	Abcam	Both 1∶100

### Serology

Gold standard serological diagnosis for scrub typhus was performed using paired serum samples with the indirect microscopic immunofluorescence assay for detection of IgM, with a cut-off titer of 1∶400 and a four-fold increase on paired serum samples, using pre-coated microscopic slides with antigen derived from validated *O. tsutsugamushi* type-strains (Karp, Kato and Gilliam), prepared and provided by the Australian Rickettsial Reference Laboratory [21]. Region-specific and previously determined positivity criteria of 1∶400 IgM or a fourfold (or greater) rising titer was taken as indicative of an active infection [4], [22].

### Quantitational PCR assay

Samples were examined by a previously described method using quantitative realtime PCR assay based on the *groEL* gene to confirm infection during diagnosis, and to quantitate rickettsial DNA copy numbers in samples from peripheral blood buffy coat preparations and skin biopsy sections [23]. Diagnostic positivity was confirmed using a nested PCR technique for the 56 KDa surface protein as previously described [24].

### Sample preparation for Electron Microscopy

Cell culture pellets or skin biopsies for EM examination were fixed in 4% glutaraldehyde in 0.1 M phosphate buffer and stored at 4°C before use. The samples were post-fixed in 2% osmium tetroxide in phosphate buffer and dehydration in ethanol followed by treatment with propylene oxide, prior to embedding in Spurr's epoxy resin. 1 µm ultrathin sections were cut with a diamond knife ultratome, stained with Azure A and examined by light microscopy to identify areas of interest. Thin sections of suitable areas were examined using a Jeol 1200EX electron microscope.

### *In vitro* human cell line cultures

U937 immortilised human cell lines were used as a model of cells with the characteristics of monocyte/macrophages [28]. Cells were grown in suspension in antibiotic free RPMI+10% FCS+1 mM Glutamine. Infection with *O.tsutsugamushi* strain UT-76 was achieved by a multiplicity of infection (MOI) of 10∶1. The infected VERO cells were incubated at 37°C for 45 minutes to then be harvested for PCR, electron microscopy and immunohistochemistry.

### Histology

Skin biopsies were immediately fixed in 10% PBS buffered formalin, stored at 4°C and subsequently processed into paraffin wax blocks. These were prepared using standard histological techniques, and 3 µm sections cut with a rotatory microtome either onto standard glass slides (for histology) or Vectabond coated slides (Vector Laboratories, Inc., Burlingame, CA, USA) for immunohistochemistry or immunofluorescence.

### Immunohistochemistry

Sections were de-waxed with Citroclear (TCS Biosciences, Buckingham, UK): 2 baths of each 5 min, and ethanol: 2 baths of each 2 min in 100% EtOH then one bath for 2 min in 50% EtOH. The sections underwent antigen retrieval by pressure cooking in Tris-EDTA buffer (pH 9), at 120°C for 2 min before double-immunological labelling.

### Immunoenzymatic staining

The DAKO EnVision™+ System, HRP kit was used. Briefly, endogenous peroxidase activity was blocked by incubating the specimen for 5 minutes with DAKO Peroxidase Block (included in the kit). Slides were incubated at room temperature in a dark, humidified chamber with primary mouse or rabbit antibody for 30 minutes or, as a negative control, incubated with PBS with omission of the primary antibody, followed by three washes with PBS and then incubation with the labeled polymer for 30-minutes. Staining was completed by a further three washes with PBS andd then a 5–10 minute incubation with 3,3′-diaminobenzidine (DAB)+ substrate-chromogen, which resulted in a brown-coloured precipitate at the antibody binding site. Double immunolabelling with immunoperoxidase staining utilized secondary labeled antibodies against different isotype specific primary monoclonals, one for which labeled with Diaminobenzidine (DAB) in brown and the other with alkaline phosphatase (AAP) in red to allow co-localisation. This was followed by counterstaining with hematoxylin for 30 seconds, washing with PBS, application of mounting media (VectaMount medium, Vector Labs, Burlingame, USA) and coverslipping the slides.

### Immunofluorescence and confocal microscopy

The 3 µm thin sections underwent pressure cooking for antigen retrieval, were incubated with the proprietory protein blocking solution (DAKO) for 10 minutes, washed three times with 10% PBS, then incubated in a humidified darkened slide chamber at room temperature with primary monoclonal antibodies (mAbs) for 30–45 min, washed and incubated with the secondary isotype specific mAbs conjugated with a fluorochrome for 30 mins. The fluorochromes used for detection of *O. tsutsugamushi* was FITC- (absorbtion 488 nm/emission 520 nm, bright apple green colour) and for cellular phenotyping Texas Red (absorbtion 596 nm/emission 615 nm, deep red colour). These were then washed in a PBS bath for 2 minutes, the slides mounted and coverslipped in VectaMount (Vector Laboratories, Burlingame, CA, USA) into which was added the nuclear intercalating counterstain DAPI (4′,6-diaminidimo-2-phenylindole), and examined using standard fluorescence microscopy and a confocal laser scanning microscope (Zeiss LSM 700). Both microscopes used the AxioVision 40 v4.7.1.0 software from Carl Zeiss Imaging Solutions Gmbh, Germany. Images were merged and optimised with Photoshop CS3 extended, version 10.0.

### Generation of anti-*O.tsutsugamushi* monoclonal antibodies

The in-house monoclonal antibody used for staining *O. tsutsugamushi* in this study was generated in our laboratory by immunization of mice using whole protein extracts of *O. tsutsugamushi* (Karp UT76, Kato, Gilliam and 716-like strains), which were cultured in L929 cells, or intra-peritoneal mouse infection with live *O. tsutsugamushi* (MSc thesis of Ampai Tanganuchitcharnchai, Faculty of Tropical Medicine, Mahidol University, Thailand). Prospective hybridomas were further chartacterised by indirect ELISA and immuno-fluorescence assays (IFA) using a panel of crude surface membrane lysates including *O. tsutsugamushi* (Karp, Kato, Gilliam and TA716-like strains), *R. typhi*, *R. prowazekii*, *R. honei*, *R. conorii*, and a wide range of negative control bacteria including *Aeromonas hydrophilia* (American Tissue Culture Collection reference 4965), *Escherichia coli* (ATCC 25922), *Shigella flexneri* (ATCC 12022), *Plesimonias shigelloides* (ATCC 14029), *Salmolella choleraesuis* (ATCC14028), *Vibrio* spp. (ATCC33812), *Salmonella* spp, *Yersinia enterocolitica*, *Pseudamonas aeruginosa* and *Leptospira* spp. Selected clones showing positive reaction specifically to *O.tsutsugamushi* were tested using Western blotting on SDS-PAGE gels of crude protein extracts of VERO infected cells. Eight mAbs were assessed for their suitability for immunostaining of cell culture preparations by IFA staining of infected and uninfected Vero cell culture pellets as positive and negative controls performed in triplicate and immuno-enzymatic staining on cell cultures (HRP-based enzyme-conjugated polymer kit, Envision, DAKO). The antibody clone 1C4B11 was a highly *O. tsutsugamushi*-specific monoclonal antibody (isotype IgG2bκ) recognizing the 56 kDa surface antigen on Western blotting, which stained *O. tsutsugamushi* strongly and specifically as ringforms with a central lucency, reflecting recognition of antigen on the outer membrane, with no staining of uninfected VERO or L929 cell lines as negative controls. It also produced regular and highly specific staining of *O. tsutsugamushi* in formalin fixed sections using AAP, HRP enzymatic and fluorescence labeling.

### Other antibodies used for immunohistochemistry

The cell-specific monoclonal antibodies used to characterize and immunophenotype different host cells within eschar biopsies are summarized in Table 1. Staining of the cellular components in sections was expressed as a percentage of all inflammatory cells. The extent of immunopositivity in the inflammatory cells for specific cell markers was graded in the following manner: (−) in 0%, (±) in <1%, (+) in 1%–25%, (++) in 26%–50%, (+++) in >50% and (++++) in >75% of infiltrated cells.

## Results

### Clinical details of scrub typhus patients

Eschar biopsies from patients with a confirmed diagnosis of scrub typhus were examined by histology, immunohistochemistry, immunofluorescence and electron microscopy (Table 2).

**Table 2:** Overview of patient and eschar characteristics.

Patient TM Nr.	Days Fever	Age	Eschar Location	Lymph-adenopathy	Bacteria in eschar	Days in Hospital	IgM – Serology (reciprocal titers)		Bacterial load in blood (DNA copies/mL)	
							Adm	FUP	Adm	FUP
2193	5	64	Abdomen	Neg	+++	20	3200	≥25600	2060	Neg
2508	7	30	Abdomen	Pos	+++	5	200	3200	3665	Neg
2425	7	25	Axilla	Pos	++	10	200	≥25600	1220	Neg
2644	7	60	Abdomen	Neg	+	6	100	800	Neg	Neg
2391	10	30	Head	Neg	+	7	1600	6400	Neg/850	Neg
2663	11	28	Arm	Neg	0	5	1600	≥25600	Neg	Neg
2646	12	66	Abdomen	Neg	0	8	3200	NA	3270	Neg
1179	10	25	Groin	Pos	EM	15	6400	≥25600	960/Neg	Neg
1317	7	35	Arm	Pos	EM	4	≥25600	≥25600	1640	Neg
1242	7	43	Gluteus	Neg	EM	8	≥25600	≥25600	1120	Neg
1320	7	20	Chest	Pos	EM	7	50	≥25600	2720	Neg

Summary of basic clinical patient data and eschar characteristics by patient [TM = Typhus Mahosot], days of fever prior to biopsy [days], patient age [years], eschar location, presence of clinical lymphadenopathy, bacterial number of *O. tsutsugamushi* in eschar as defined by microscopy [semi-quantitative scale representing bacterial count per microscopic field at ×400 magnification; (0) = absent no bacteria/field, (+) = mild <25 bacteria/field, (++) = moderate 25–50/field, (+++) = extensive>50/field], admission and follow-up serology [reciprocal anti-*O. tsutsugamushi* IgM antibody titers] and bacterial load on quantitative realtime PCR [mean DNA copy numbers per mL serum]. EM = electron microscopy sample; NA = not available.

All patients presented with fever and suffered from headache or severe retro-orbital pain (10/11), myalgia (10/11) or rigors (10/11). No skin rash was observed in any of the patients. One patient presented with confusion and deafness, which resolved fully following antibiotic therapy. Four patients were referred after β-lactam based antibiotics had been administered with no effect. All patients were subsequently treated with doxycycline, with exception of a pregnant woman, who was treated successfully with azithromycin. No patients suffered severe manifestations of the disease and all made a full recovery during hospitalization [median 8 days (range 4–20)].

### Histological features of eschar biopsies

**Figure 1:**
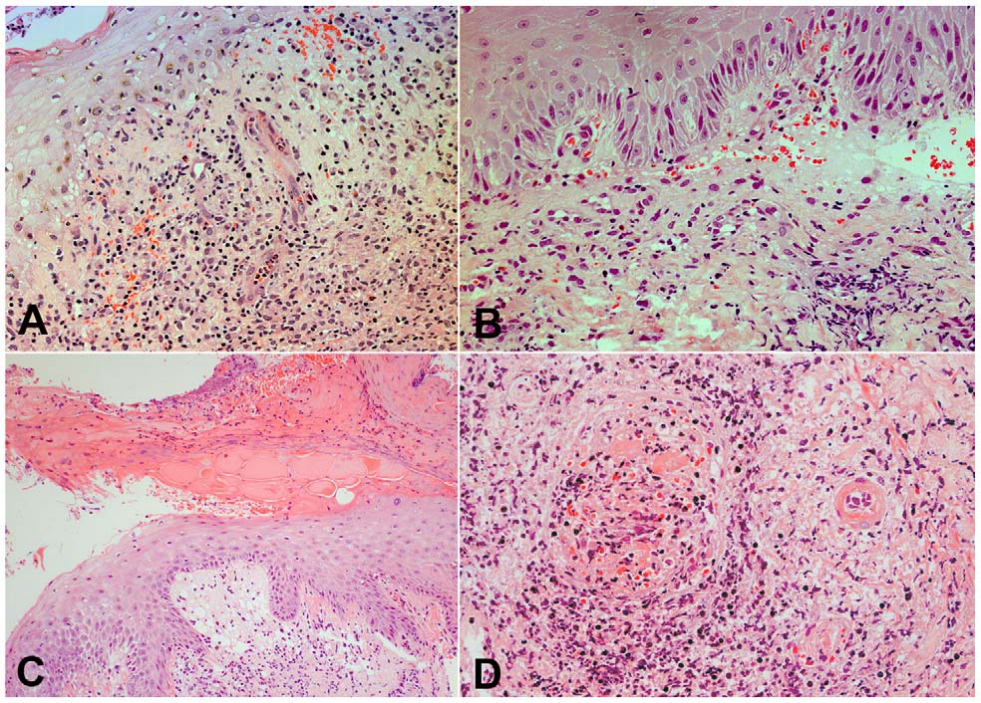
Histological features of eschars: Intra- and subepidermal changes. Panels A and B. Intraepidermal splitting and sub-epidermal blistering with basal vacuolar changes, inflammatory exocytosis and focal red blood cell extravasation in the superficial dermis. Patient TM2644 (Panel A) and TM2646 (Panel B), magnification ×400. Panel C. Acute inflammatory sloughing and artefactual separation of the epidermis. A demarcated area of ulceration and necrosis of the overlying epidermis with subepidermal vacuolation, necrosis and mononuclear infiltration, patient TM2663, magnification ×250. Panel D. Perivascular infiltrates and leucocytoclastic vasculitis. On the right hand side the cross-section of an arteriole, on the left, a venous vessel with intravascular obliteration and secondary thrombus. There is prominent perivascular cuffing with mononuclear cells and fibrinoid necrosis of the arteriolar wall. Patient TM2193, magnification ×250. All panels Haematoxylin & Eosin (H&E) staining.

Histological examination of the eschar revealed common features between patients, with sharply demarcated areas of ulceration and necrosis of the overlying epidermis, acute inflammatory slough and artifactual shedding and blistering of the epidermis (Figure 1). The neighbouring intact epidermis showed mild spongiosis with focal inflammation. There was focal parakeratosis, probably reflecting the relatively short time period over which the eschar is established. Basal vacuolar and necrotic changes within keratinocytes were accompanied with focal red cell extravasation, and in patient TM2644 there was established intraepidermal splitting, and in patient TM2646 marked subepidermal blistering. These features appeared secondary to necrosis, and associated with marked neutrophilic acute inflammation, rather than representing a primary bullous disease [25].

In the underlying dermis the histological pattern was dominated by inflammatory infiltrates at all levels, including perivascular infiltrates in subdermal fat consistent with superficial panniculitis. The dermis showed mild edema and marked mononuclear cell infiltrates, distributed in perivascular, interstitial, perifollicular, periglandular and focally in perineural areas. Patient TM2193 showed a leucocytoclastic vasculitis, with dense infiltrates of mononuclear cells around dermal vasculature, karryorhectic neutrophil debris, fibrinoid necrosis of the vessel wall and associated thrombosis.

### Immunophenotyping of leucocyte subsets in eschar infiltrates

**Figure 2:**
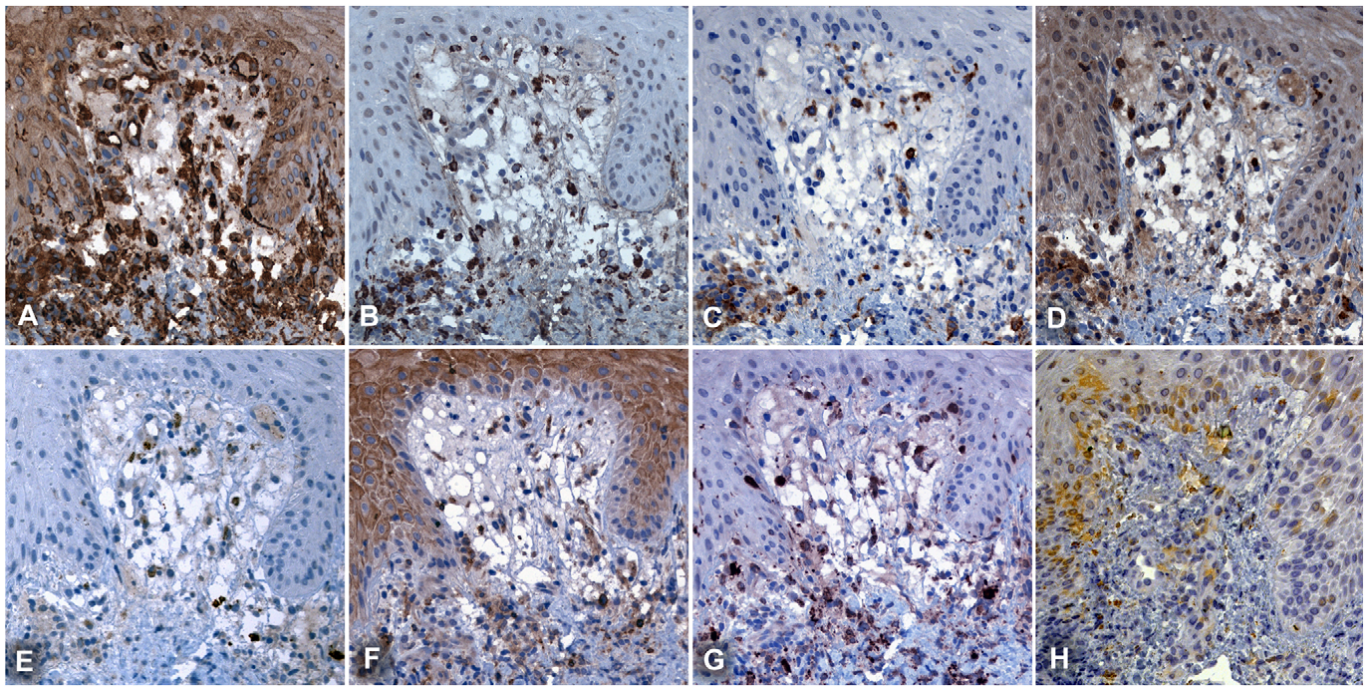
Immunohistochemical phenotyping of cellular infiltrates at the dermal-epidermal border. Serial sections at the dermal-epidermal border, demonstrate that APCs are the dominant cell type adjacent to the central necrotic zone of the eschar. Immunophenotyping by MHC class II receptor for APCs (Panel A: HLADR), macrophages (B: CD68) and DCs (C: DC-SIGN, D: FXIIIa, G: S100 and H: LSECtin), neutrophils (E: CD15) and lymphocytes (F: CD4). The proportion of dendritic cells was higher in the dermal-epidermal border zone, than in the deeper dermis. Subepidermal vacuolization is prominent. Patient TM2663, magnification ×400, counterstained with haematoxylin, peroxidase immunostaining in brown.

The dermal cell infiltrates were phenotyped using multiple markers against different leucocyte subsets as detailed in Table 1. A mixed inflammatory infiltrate with a strong predominance of antigen presenting cells (APCs) was present within all the eschar biopsies studied. The extensive infiltrates, in both superficial and deep dermis, consisted predominantly of mononuclear cells with high expression levels of MHC class II receptors (HLADR). High leucocyte densities were found at the dermo-epidermal junction, around vessels and sebaceous and sweat glands reaching into the subcutaneous fat (Figure 2). More detailed examination of HLADR intense zones in the eschar revealed that more than 80% of these cells consisted of monocytes, macrophages and dendritic cells (Supplemental Figure S1). Lymphocytes were found interspersed and whilst T cells were common, B cells were rare. T lymphocytes presented with a ratio of T helper cells (CD4+) to cytotoxic T cells (CD8+) of approximately 1∶2. The presence of dermal dendritic cells (DDCs) was more intense at the dermal-epidermal junction, with a macrophage to DDC ratio of approximately 1, which increased towards the deeper dermis. DDCs displayed an immunophenotype of CD1a+, CD11c+, DCSIGN+, S100+, and FXIIIa+ (using double immunofluorescence labelling on multiple sequential sections). Neutrophils were relatively uncommon in perivascular infiltrates but formed an intense zone of infiltration around the necrotic centre of the eschar (Supplemental Figure S2).

### Characterization of *O. tsutsugamushi*-infected cells in eschars

#### Endothelial cells

Intracellular infection of endothelial cells by *O. tsutsugamushi* in the dermal vascular plexus of these specimens was extremely rare and found in only three sections (patients TM2193 and 2508). While *O. tsutsugamushi* co-localization with the endothelium was often seen, the high resolution thin sections using confocal laser scanning microscopy (LSM) revealed *O. tsutsugamushi* to be located within adjacent perivascular mononuclear cells or cells in the process of diapedesis through the endothelial monolayer (Supplemental Figure S3).

#### Antigen presenting cells (APCs)

**Figure 3:**
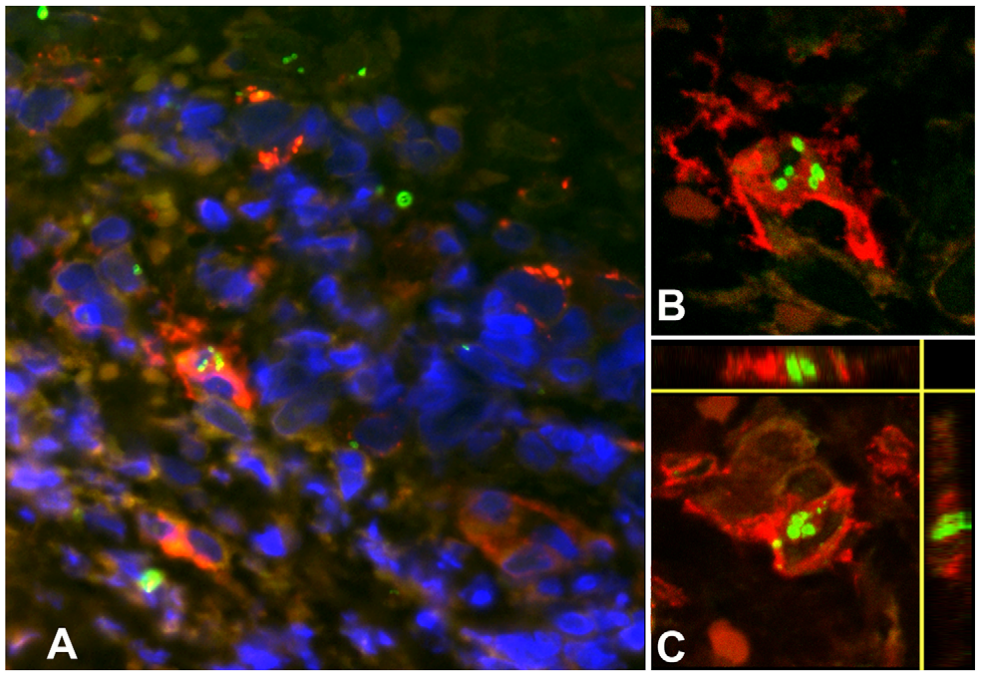
Multiple intracellular *O. tsutsugamushi* within antigen presenting cells (APCs) in the superficial dermis. APCs are characterised by MHC class II receptor (HLADR) positivity and were associated with intracellular *O. tsutsugamushi* in admission samples of an eschar from a patient with acute scrub typhus. Panel A: HLADR-positive cells in red, *O. tsutsugamushi* in green. Panels B and C: Laser Scanning Micrographs. Panel B depicts the same infected cell as in Panel A as a 0.3 µm thin section. Panel C shows a 3D stack projection (of 0.3 µm thin sections) of intracytosolic *O. tsutsugamushi* (in green), with small, high-density granules with high refractive index, staining positively for *O. tsutsugamushi* antigen. Patient TM2193, magnification ×400, LSM inserts ×1000, double-immunolabeling: HLADR in red, *O. tsutsugamushi* in green and DAPI nuclear counterstain in blue.

**Figure 4:**
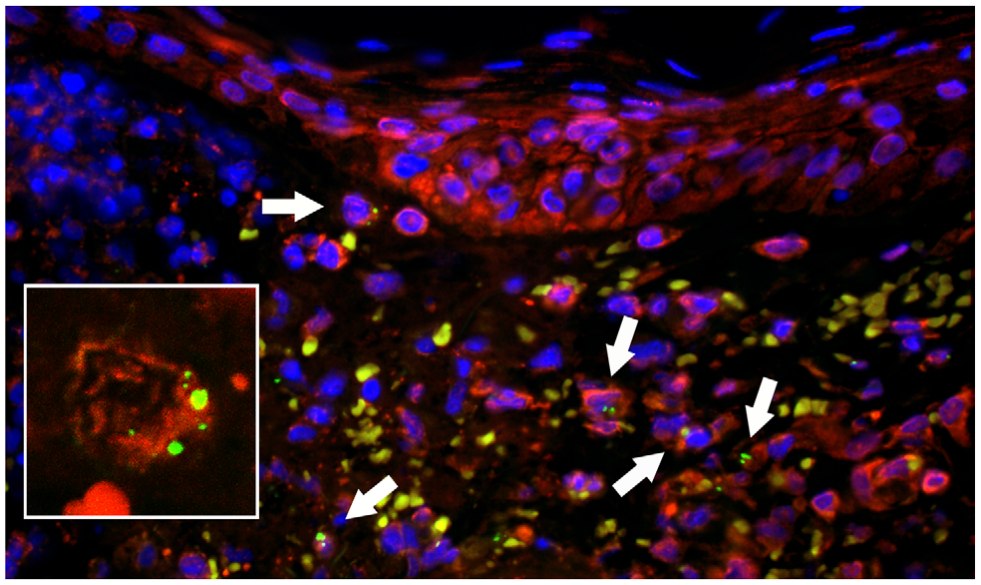
Multiple intracellular *O. tsutsugamushi* within CD1a positive Langerhans cells in the superficial dermis. Local proliferation of Langerhans cells in the epidermis, with a distinct CD1a+ nodule formation and *O. tsutsugamushi* infected cells located in the dermis (white arrows) of an eschar from a patient with acute scrub typhus. Erythrocytes appear yellow due to autofluorescence. Insert: Laser Scanning Micrograph (LSM), 0.3 µm thin section visualizing the intracytosolic location of *O. tsutsugamushi*, accompanied by small intracellular microparticles with high refractile index. Patient TM2193, magnification ×400, insert ×1000. Labeling: CD1a in red, *O. tsutsugamushi* in green and DAPI nuclear counterstain in blue.

**Figure 5:**
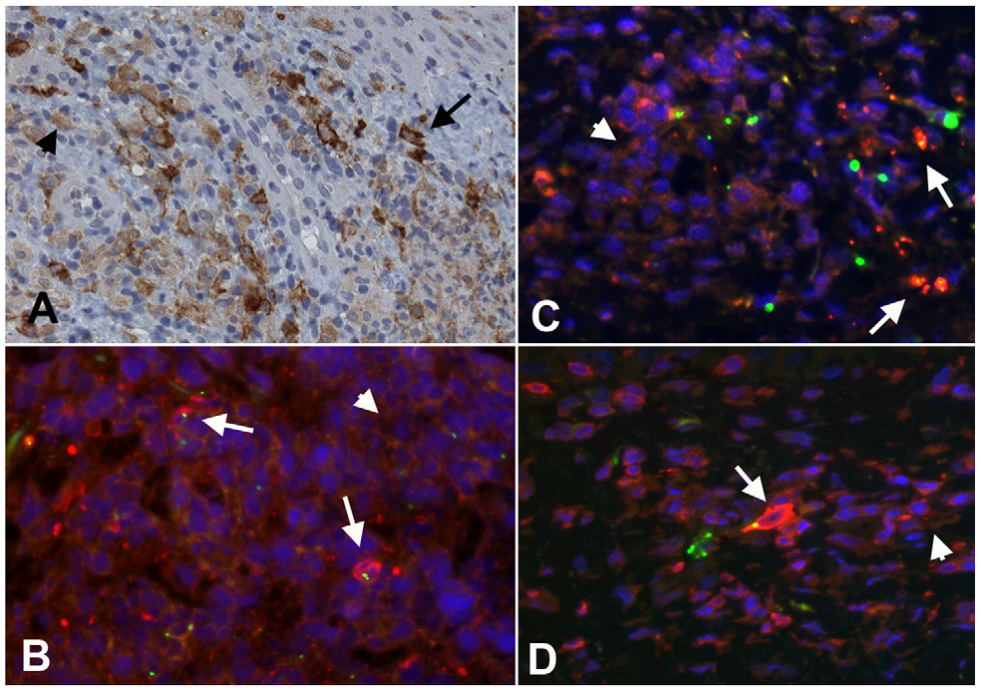
Dermal dendritic cells in the dermal-epidermal zone of the eschar. Panel A: High numbers of DCSIGN+ cells in the dermal-epidermal zone of the eschar. The variance of staining-intensity of individual positive cells ranged from strong (long black arrow) to weak (short black arrow). Patient TM2644, magnification ×400, peroxidase immunostaining in brown. Panels B, C and D: Immuno-double-labelling with HLADR (Panel B), DC-SIGN (Panel C) and LSP-1 (Panel D) stained red. The signal intensity of these markers in infected cells with *O. tsutsugamushi* (in green) appeared more pronounced (long white arrows) than in uninfected cells (short white arrows). Patient TM2193 (Panel B), patient TM2508 (Panel C and D). Magnification ×400, blue nuclear counterstaining with DAPI.

APCs, characterized by MHC class II receptor and HLADR positivity, were the main cell type associated with intracellular *O. tsutsugamushi* infection (Figure 3). HLADR positive cells contained high numbers of *O. tsutsugamushi*, (6–7 organisms per cell per section), but also small immuno-positive intracellular microparticles, with high refractive indices on confocal LSM. This ‘microparticle’ phenomenon could not be observed in any other of the cell types characterized. HLADR-positive dermal dendritic cells (DDCs) cells were differentiated from monocyte/macrophages by their CD1a, S100, LSECtin and/or DC-SIGN positivity. DDCs were the predominant HLADR+ cell type found in the sub-epidermal zone. A focal subpopulation of CD1a-positive Langerhan's cells infected with *O. tsutsugamushi* were seen entering the dermis in conjunction with overlying nodule-like epidermal proliferations of Langerhan's cells (Figure 4). This was more pronounced in immediate vicinity of the necrotic zone. Although CD1a is a hallmark antigen for Langerhan's cells, these surface markers can also be found on activated lymphocytes, and DDCs [26], [27]. DCSIGN+ cells were present in large numbers in the dermis, and two subsets of DDCs were characterized by either weak or strong intensity, in both immunoperoxidase and immunofluorescence staining. LSM images demonstrated intracellular *O. tsutsugamushi* within DDCs, and a strong DCSIGN+ signal was observed with evidence of *O. tsutsugamushi* infection in the same cell, as shown by co-localization with *O. tsutsugamushi* in LSM thin sections (Figure 5). Similar observations were made for subsets of LSECtin+ and S100+ DCs (data not shown).

**Figure 6:**
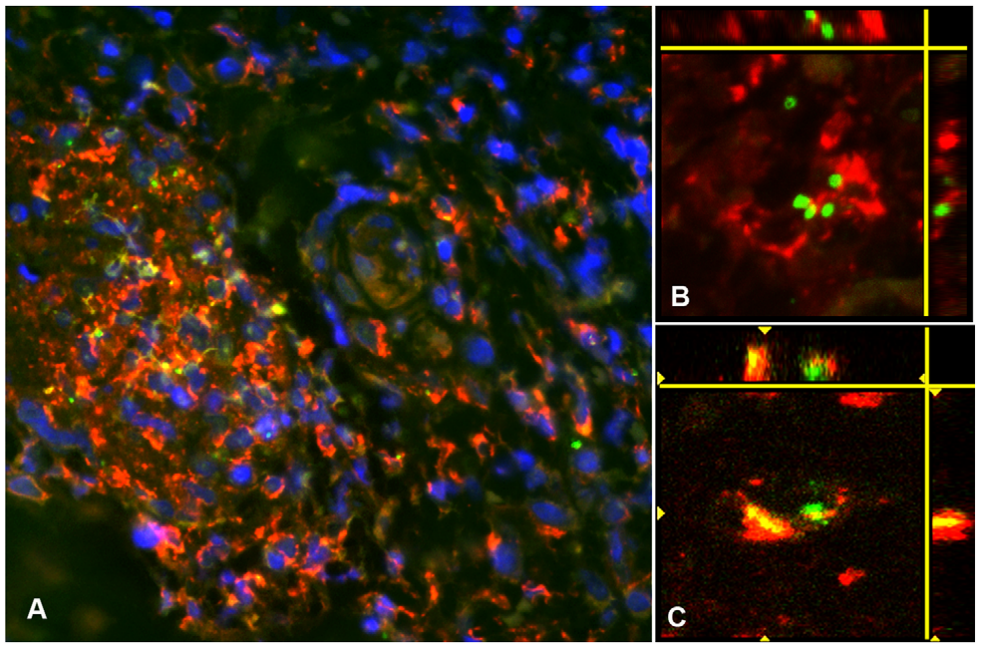
*O. tsutsugamushi* co-localize with CD68 positive macrophages in perivascular infiltrates. Panel A: Perivascular cuff formation and clusters of predominantly CD68+ macrophages, co-localizing closely with *O. tsutsugamushi*. Patient TM2193, magnification ×400. Magnification ×400, insert ×1000, double-immunolabeling: CD1a in red, *O. tsutsugamushi* in green and DAPI nuclear counterstain in blue. Panels B and C: Laser Scanning Micrographs with 3D stack projections of multiple 0.3 µm thin sections, depicting intact *O. tsutsugamushi* associated with CD68+ macrophages (panel B). Unambigous intracellular location of *O. tsutsugamushi* in a CD68+ cell (panel C). Patient TM2508, magnification ×1000, CD68 labeled in red, *O. tsutsugamushi* in green.

**Figure 7:**
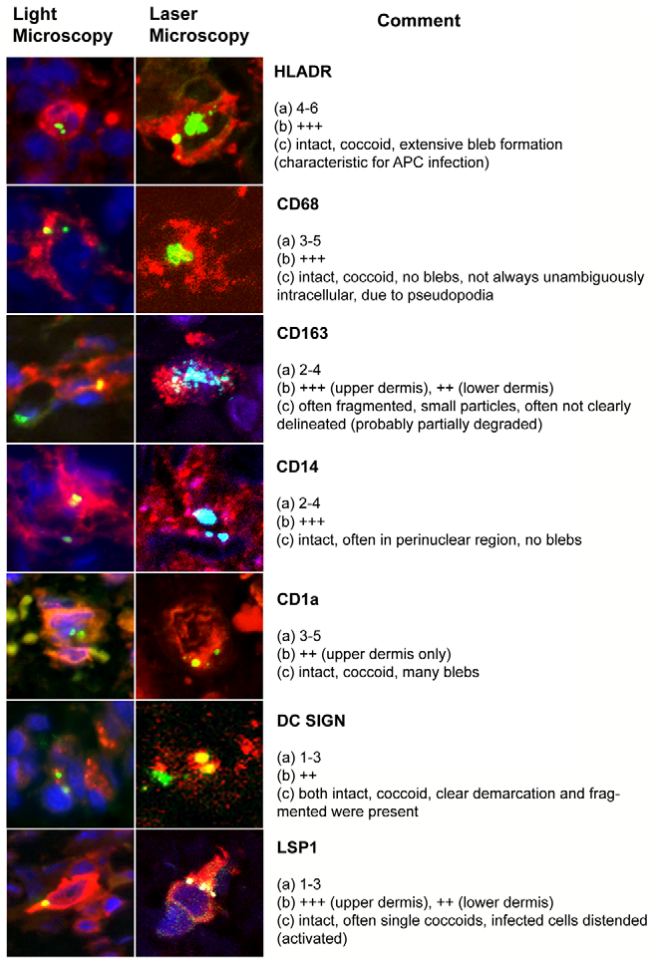
Overview of host-cell phenotypes associated with *O. tsutsugamushi* infection in vivo in human eschars. Characteristics were defined as follows: (a) Number of *O. tsutsugamushi* per cell per section. (b) Proportion of cells in infiltrate. (c) Morphological aspects of intracellular *O. tsutsugamushi*. Infiltrates were estimated in a semi-quantitative manner: The extent of immunopositivity in the inflammatory cells for specific cell markers was graded in the following manner: (−) in 0%, (±) in <1%, (+) in 1%–25%, (++) in 26%–50%, (+++) in >50% and (++++) in >75% of infiltrated cells.

**Figure 8:**
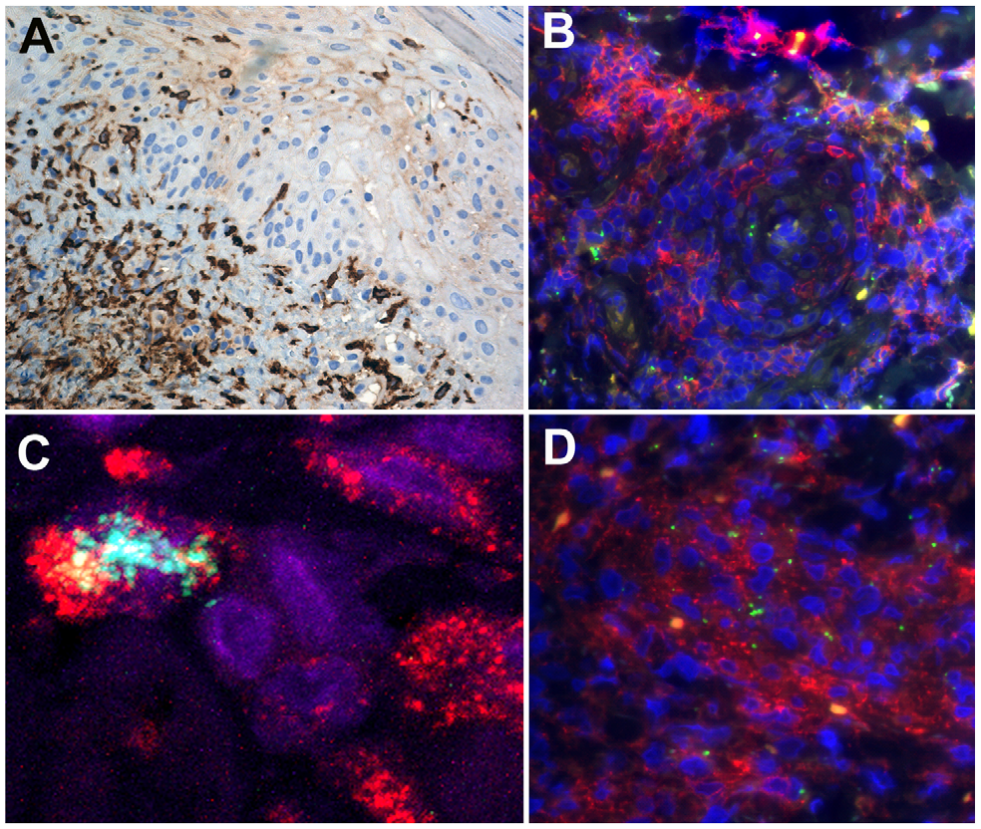
*O. tsutsugamushi*-infected monocytes/macrophages in dense perivascular infiltrates express CD14 and scavenger receptor CD163. Panel A: High density of CD163+ monocye/macrophages at the dermal-epidermal border and after intraepidermal translocation adjacent to the inoculation of *O. tsutsugamushi*. Patient TM2391 (panel A), magnification ×200, peroxidase immunostaining in brown. Panels B and D: Perivascular CD14+ monocyte/macrophages conglomerates containing *O. tsutsugamushi*. Cross-section of perivascular cuff formation (panel B) and longitudinal section (panel D). Patient TM2644, magnification ×400, double-immunolabeling: CD14 in red, *O. tsutsugamushi* in green and DAPI nuclear counterstain in blue. Panel C: Laser Scanning Micrograph, 0.3 µm thin section confirms the intracellular co-localization of *O. tsutsugamushi* in a CD163+ monocyte/macrophage. A large proportion of *O. tsutsugamushi* in these cells appeared in a fragmented state. Patient TM2508, magnification ×1000, double-immunolabeling: CD163 in red, *O. tsutsugamushi* in green and DAPI nuclear counterstain in blue.

Characterisation of monocytes/macrophages included immunophenotyping for CD14, CD68 for moncytes and macrophages and CD163 for subtypes expressing scavenger receptors. Monocytes and macrophages were the predominant cell type characterizing peri-glandular and peri-vascular dermal infiltrates and co-localized regularly with *O. tsutsugamushi*, however unambiguous intracellular location could not always be proven due to podocyte formation and dendritic cellular morphology (Figure 6). Infected cells contained on average fewer intracellular *O. tsutsugamushi* than DDCs (2–4 organisms per cell per section, Figure 7). In CD163+ macrophages, the *O. tsutsugamushi* often appeared fragmented and not as round intact bacteria (Figure 8). No intracellular microparticle formation was observed in macrophages. Bacterial staining in association with B cells (positive for CD20 and nuclear BCL11a) was extremely rare and seen in only 2 individual B-cells (<1%).

#### Non-antigen presenting cells

**Figure 9:**
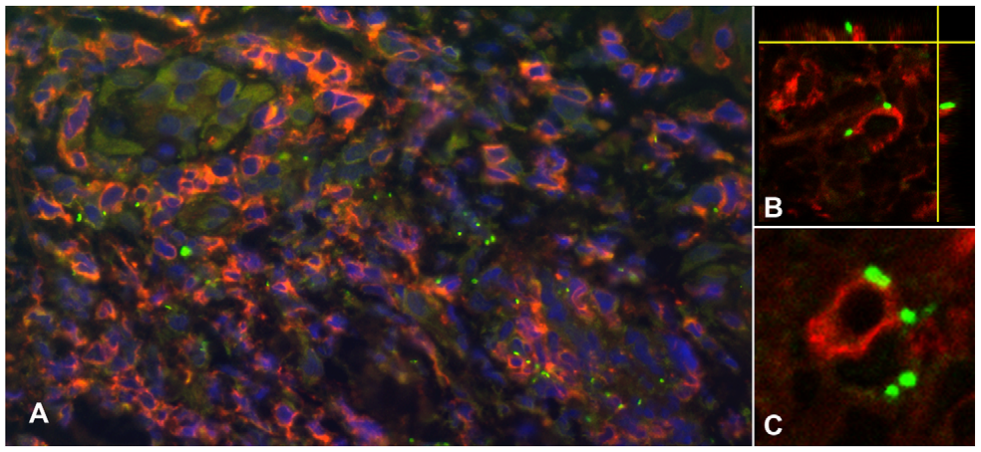
The co-localisation pattern of *O. tsutsugamushi* with lymphocytes. Panel A: Perivascular cuff formation of predominantly mononuclear cells containing large numbers of CD3+ cells co-localising with *O. tsutsugamushi*. Panels B and C: Laser Scanning Micrographs. The association of T cells with *O. tsutsugamushi* was commonly by external attachment rather than definite intracellular location. Panel B depicts a 3-D stack projection showing external *O. tsutsugamushi* attachment to the T lymphocyte, in the lateral vertical sections. Panel B is a 0.6 µm thin section. Patient TM2193, magnification in panel A ×400, in panels B and C ×1000. Double-immunolabeling: CD3 in red, *O. tsutsugamushi* in green and DAPI nuclear counterstain in blue.

CD3+ T lymphocytes co-localized with *O. tsutsugamushi* and confocal LSM revealed the external attachment of *O. tsutsugamushi* to be more common than true intracytoplasmic infection (Figure 9). CD3 stains both CD8+ cytotoxic T-cells (MHC class I) and CD4+ regulatory T-cells (MHC class II), but immuno-fluorescence staining for CD8+ T-cells showed co-localization in only 3 cells (<1%).

LSP1 is expressed in lymphocytes, neutrophils, macrophages, and endothelium and may regulate neutrophil motility, adhesion to fibrinogen matrix proteins, and transendothelial migration. LSP1 can also be expressed by plasma cells, dendritic cells and Langerhans cells [28]. O. tsutsugamushi co-localized intracellularly with LSP1+ cells in the upper dermis and infected cells appeared to stain more intensively for LSP1 (Figure 5).

Neutrophils were most dense at the delineating zone of the central necrosis and CD15/neutrophil elastase-positive cells co-localized regularly with intracytoplasmic *O. tsutsugamushi*. While many bacteria appeared intact, a proportion appeared to vary in size and morphology, suggestive of intracytoplasmic degradation.

#### Quantitation of bacterial loads and leucocyte responses

As summarized in Table 2, semiquantitative estimation of bacterial load within the eschar showed high levels of infected cells in eschars taken earlier in the course of febrile illness (days 5–7) and decreased (or absent) *Orientia* seen in later eschars from days 7–12. Bacterial load per infected cell ranged from 1–7 recognisable separate bacteria per section. Semiquantitative estimation of specific infected cell phenotypes showed activated inflammatory monocytes and dendritic cells were present in both the upper and lower dermis, although the nodules of CD1a+ Langerhan's cells were limited to the basal epidermis and superficial dermis. Quantitative PCR estimation of bacterial load in peripheral blood leucocytes from Buffy coat samples indicated that early dissemination of *Orientia* into the blood stream was already established at presentation of the eschar. The decrease in bacterial numbers in the eschar over time (taking the series as a whole) and the early positive PCR results in Buffy coat samples imply that the eschar is not the major site for bacterial replication.

#### Formation of *Orientia* membrane protrusions

**Figure 10:**
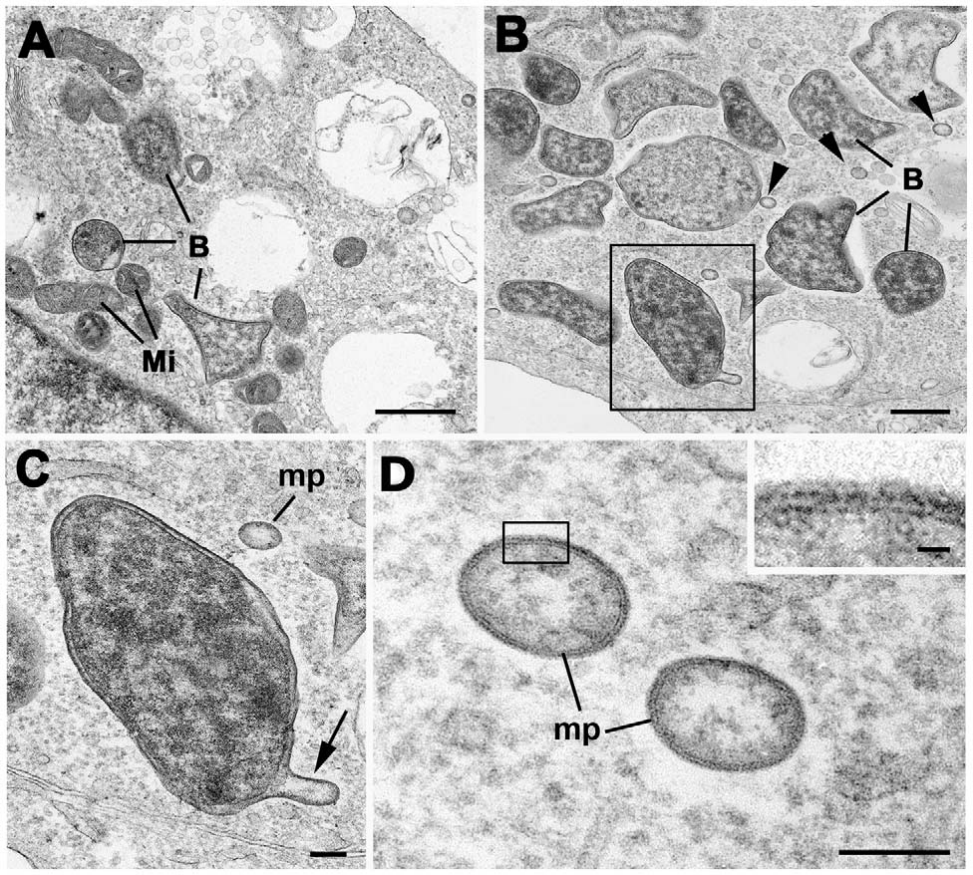
Ultrastructural examination of *O. tsutsugamushi* in U937 cells reveals microparticle formation. Panel A: Bizarre morphology of *O. tsutsugamushi* (B) in the U937 cell line, with corners and tent-like stretching within the cytoplasm with adjacent mitochondria (Mi). Bar length corresponds to 1 µm. Panel B: A number of *O. tsutsugamushi* (B) with associated microparticles (arrowheads) within the host cell cytoplasm. Bar represents 500 nm. Panel C: Enlargement of the enclosed bacterium from Panel B showing blebbing of the outer membrane (arrow). ‘mp’ - detached microparticle. Bar length corresponds to 100 nm. Panel D: Detail of two microparticles (mp) formed by the outer, but not inner leaflet of *O. tsutsugamushi*. These microparticles carry the surface antigens of the ‘mother’ *Orientia*, and their function remains unclear. Bar length corresponds to 100 nm. Insert - Enlargement of the enclosed area in D showing the structure of the limiting membrane of the microparticle. Bar length corresponds to 10 nm.

Ultrastructural examination of *in vitro O. tsutsugamushi*-infected U937 monocytic cells revealed formation of multiple intracytopalsmic membrane protrusions from bacteria, leading to microparticles budding from the outer leaflet of normal-sized, intracellular bacteria were found in in the cytoplasm of infected U937 cells (Figure 10). This mirrored morphological observations in both HLADR and CD1a positive cells in eschar biopsies *in vivo*.

## Discussion

Evidence from post mortem studies and extrapolation of findings in other rickettsial diseases have implied that scrub typhus is predominantly an endothelial infection, with pathological features of a disseminated vasculitis. Early histopathological studies from the 1940s suggested different pathogenic mechanisms between *Orientia* and *Rickettsia* spp. [29] and recent clinico-pathophysiological studies in Southeast Asia highlighted marked differences in the expression patterns of endothelial and mononuclear cell activation markers, as well as coagulation parameters and cytokines for the clinically similar diseases murine typhus (caused by *Rickettsia typhi*) and scrub typhus (*O. tsutsugamushi*) [3], [30]. While murine typhus patients had high levels of endothelial-specific soluble E-selectin, implying widespread endothelial cell activation, this was not the case in scrub typhus, where significantly raised sL-selectin levels implied mononuclear cell activation.

This study provides evidence that within the eschar site, at the clinically relevant timepoint of hospital presentation after 5 to 10 days of fever, *O. tsutsugamushi* co-localized mainly with monocyte/macrophages and dermal dendritic cells, rather than the endothelium (Figure 7). Both of these cell types act as APCs and have the ability to re-circulate systemically via the lymphatic vasculature. The *O. tsutsugamushi* organisms within these cells did not appear fragmented and formed clusters or groups, often at a perinuclear location, suggestive of intracellular replication. B-cells were very rarely infected and T-cells often showed *O. tsutsugamushi* adherent to the surface of the cell rather than invading intracellularly.

Endothelial cells on the other hand were not a common site for cellular invasion and replication and seemed to be a less important target than mononuclear cells in this early phase of infection in the eschar. A recent report demonstrated that monocytes are activated *in vitro* and *ex vivo* following exposure to *O. tsutsugamushi* [31]. These pathogenic findings offer an explanation for the rapid clearance of organisms from the eschar and early hematogenous and/or lymphatic dissemination (due to re-circulation of infected cells and/or direct hematogenous dissemination), consistent with the presence of early and co-existent rickettsiaemia by PCR examination of peripheral blood.

The eschar provides a unique opportunity to study cellular interactions in humans *in vivo*, though the biopsy specimen of each patient represents a single time window, usually early in the disease course. Although data from serial sections at different timepoints from the same eschar are not available, eschars biopsied on days 5 to 7 of clinical illness had higher bacterial counts per section than those biopsied on days 10 to 12, suggestive of a gradual decline of detectable *O. tsutsugamushi* within an eschar over time. While the number of *O. tsutsugamushi* in the eschars studied decreased rapidly from day 7, rickettsaemia as measured in the buffy coat preparation of peripheral blood leucocytes from the same patients decreased around day 10 of fever/illness, and did not relate directly to the presence of *O. tsutsugamushi* in the eschar. This implies that the main site of replication of *O. tsutsugamushi* prior to dissemination is not the eschar. Regional lymphadenopathy at this timepoint is common, which coupled with the tropism of *O. tsutsugamushi* to APC in eschars, suggests that one source of replication may be within infected, recirculated APCs in the regional (and other) lymph nodes. Previous investigations of intradermal inoculations with *R. mooseri (R. typhi)* in guinea pigs demonstrated that the organisms rapidly access draining lymph nodes then sequentially appeared in spleen, kidney, and blood [32]. However if *O. tsutsugamushi* is disseminated to other organs by this time then further studies are required to determine the sites of replication and cellular tropism within other tissues, in relation to disease progression (days of fever or similar parameters). As no studies addressing these issues in eschars of SFG and TG rickettsial disease are available, direct pathogenic comparisons of infected cell phenotypes to related rickettsioses are unfortunately not possible.

A novel observation in these studies was of small membranous protrusions forming microparticles in the cytoplasm of infected monocyte/macrophages and dendritic cells, *in vivo* (CD1a+ and HLADR+) (Figures 3 and 4) and *in vitro* in the monocyte cell line U937 (Figure 10). These microparticles stained strongly positive with the monoclonal antibody targeting the surface type specific antigen p56. Similar microbial protuberances leading to shedding of microparticles have been described in salivary gland cells of the transmitting vectors (trombiculid mites) in EM studies, with an apparently similar membrane structure [33]. They may represent particles of degenerating *O. tsutsugamushi*. Other intracellular organisms with similar membrane characteristics, such as *Ehrlichia* spp. have been reported to produce membranous rounded or filamentous extensions, which can contribute to the pathogenicity of infection [34], [35].

Dermal dendritic cells comprise at least two major phenotypic populations, immature DCs expressing CD1, CD11c and CD208; and macrophages expressing DC-SIGN (CD209), CD206, CD163, and CD68 [27]. This study showed evidence of DDC subgroups with differential expression of either HLADR, DCSIGN, LSECtin, S100 and/or LSP1. *O. tsutsugamushi*-infected DDCs appeared to express higher levels of DC-SIGN, arguing either for the selection of these cells for infection, or upregulation of this marker in response to infection. The immunomodulatory effects of *O. tsutsugamushi* infection on local APCs in the eschar site could also interfere with downstream host immune responses. Activated DDCs (such as dermal Langerhans cells) can recirculate from skin to lymph nodes and represent a potential route of intracellular spread away from the eschar site, This offers an alternative explanation for rapid systemic dissemination other than interendothelial cell migration, and may not be restricted solely to *O. tsutsugamushi*, but also other rickettsioses.

These observations of the cellular tropism of *O.tsutsugamushi* in the eschar raise the question of which cellular phenotypes are infected in the lymph node and distant organs following dissemination, where endothelial cells could be a preferential site of infection. Such data are unlikely to become available from large autopsy based studies and hence the development of good animal models for scrub typhus is important.

Further investigations are warranted into the phenotypes of cells infected in the lymph node and other organs following dissemination to determine where *Orientia* invade, survive and replicate, as this will contribute to our understanding of immunopathophysiology of scrub typhus.

## Supporting Information

Figure S1 Immunohistochemical characterization of cellular phenotypes and their distribution within dermal infiltrates. Markers used to define antigen presenting cells (panel A: HLADR), monocytes (B: CD14), macrophages (C: CD68, D: CD163), neutrophils (E: CD15), dendritic cells (F: CD11c, G: DCSIGN, H: S100, and I: FXIIIa), endothelium (J: CD31), B lymphocytes (K: CD20, L: BCL11a), T lymphocytes (M: CD4, N: CD8) and cytokeratin (O: CK). While T lymphocytes were ubiquitously present, the most prominent cellular phenotype found in dermal infiltrates were APCs, consisting predominantly of macrophages and dendritic cells, but not B cells. Patient TM2425, magnification ×400, counterstain haematoxylin, peroxidase immunostaining in brown.
(TIFF)

Figure S2 Neutrophil polymorphnuclear cells delineate the central necrotic zone of the eschar. The necrotic centre (labelled ‘N’, in upper left of both panels) is delineated by a granulocyte-dense zone depicted in red, buy immunofluorescent staining for neutrophil elastase NP57 (Panel A), and CD15 (Panel B). Accumulation of CD15+ neutrophils along the vasculature (yellow arrows) can be seen in panel B. Patient TM2193, magnification ×200, erythrocytes in yellow-green, NP57 and CD15 labeled in red, DAPI nuclear counterstain in blue.
(TIFF)

Figure S3 Longitudinal section of a superficial dermal blood vessel with perivascular mononuclear cells. A longitudinal cross-section of a blood vessel with endothelium stained by CD31 (in red) and *O. tsutsugamushi* (in green). A small cluster of *O. tsutsugamushi* (long white arrow) were examined using a LSM (insert). The insert is a 0.3 LSM micrograph with CD31+ cells (short arrows) with adjacent cells containing *O. tsutsugamushi* (long white arrow) One cell contains *O. tsutsugamushi* and three small ‘bleb-like’ inclusions, typically seen in HALDR-positive cells. Patient TM2193, magnification ×400, LSM insert ×1000, double-immunolabeling: CD31 in red, *O. tsutsugamushi* in green and DAPI nuclear counterstain in blue.
(TIFF)
