# Supplementary figures and images for: Orientia tsutsugamushi in Human Scrub Typhus Eschars Shows Tropism for Dendritic Cells and Monocytes Rather than Endothelium
Source: PLoS Negl Trop Dis. 2012 Jan 10;6(1):e1466. doi: 10.1371/journal.pntd.0001466 (PMC3254662; doi:10.1371/journal.pntd.0001466)

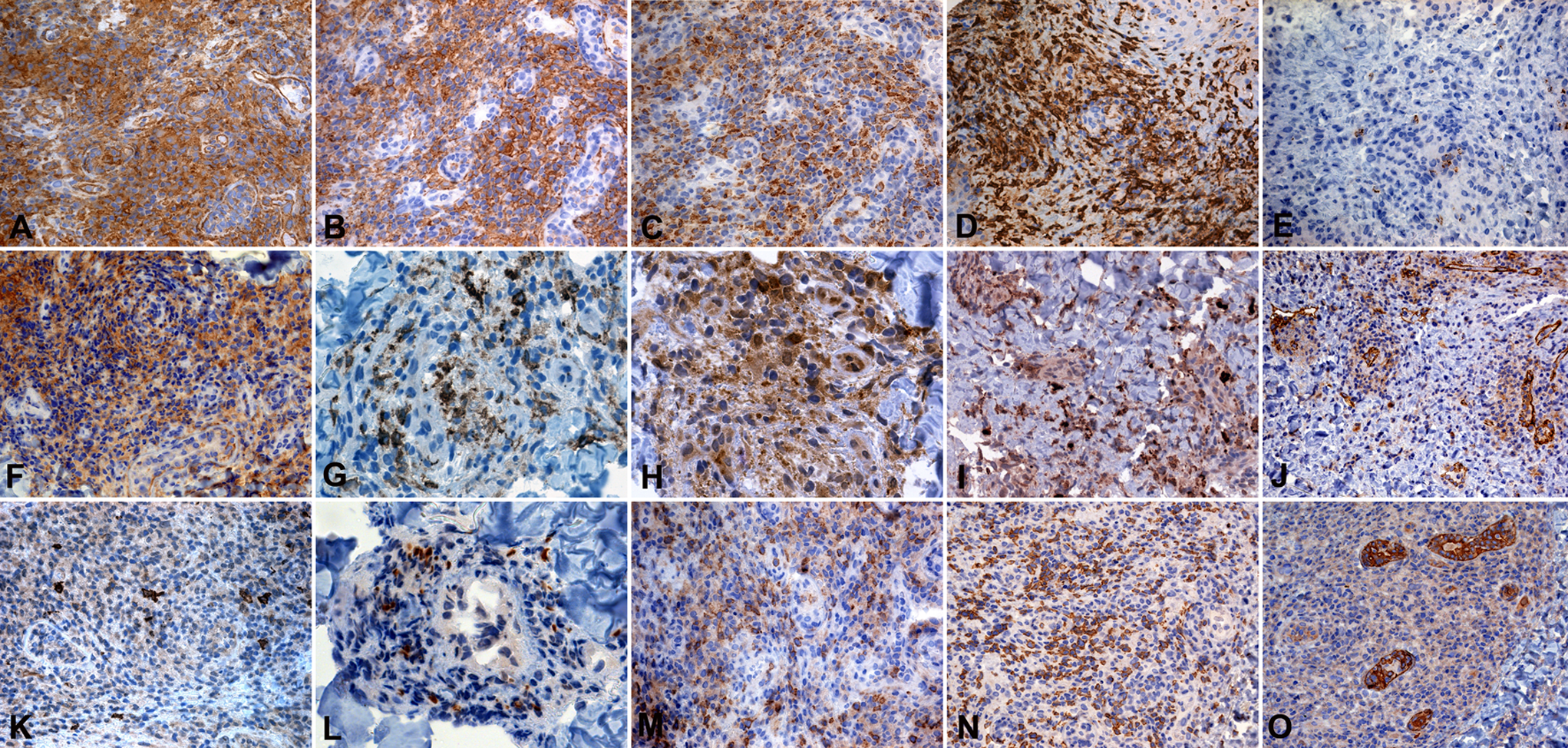

Supplement: Figure S1 — Immunohistochemical characterization of cellular phenotypes and their distribution within dermal infiltrates. Markers used to define antigen presenting cells (panel A: HLADR), monocytes (B: CD14), macrophages (C: CD68, D: CD163), neutrophils (E: CD15), dendritic cells (F: CD11c, G: DCSIGN, H: S100, and I: FXIIIa), endothelium (J: CD31), B lymphocytes (K: CD20, L: BCL11a), T lymphocytes (M: CD4, N: CD8) and cytokeratin (O: CK). While T lymphocytes were ubiquitously present, the most prominent cellular phenotype found in dermal infiltrates were APCs, consisting predominantly of macrophages and dendritic cells, but not B cells. Patient TM2425, magnification ×400, counterstain haematoxylin, peroxidase immunostaining in brown. (TIFF) [file pntd.0001466.s001.tif]

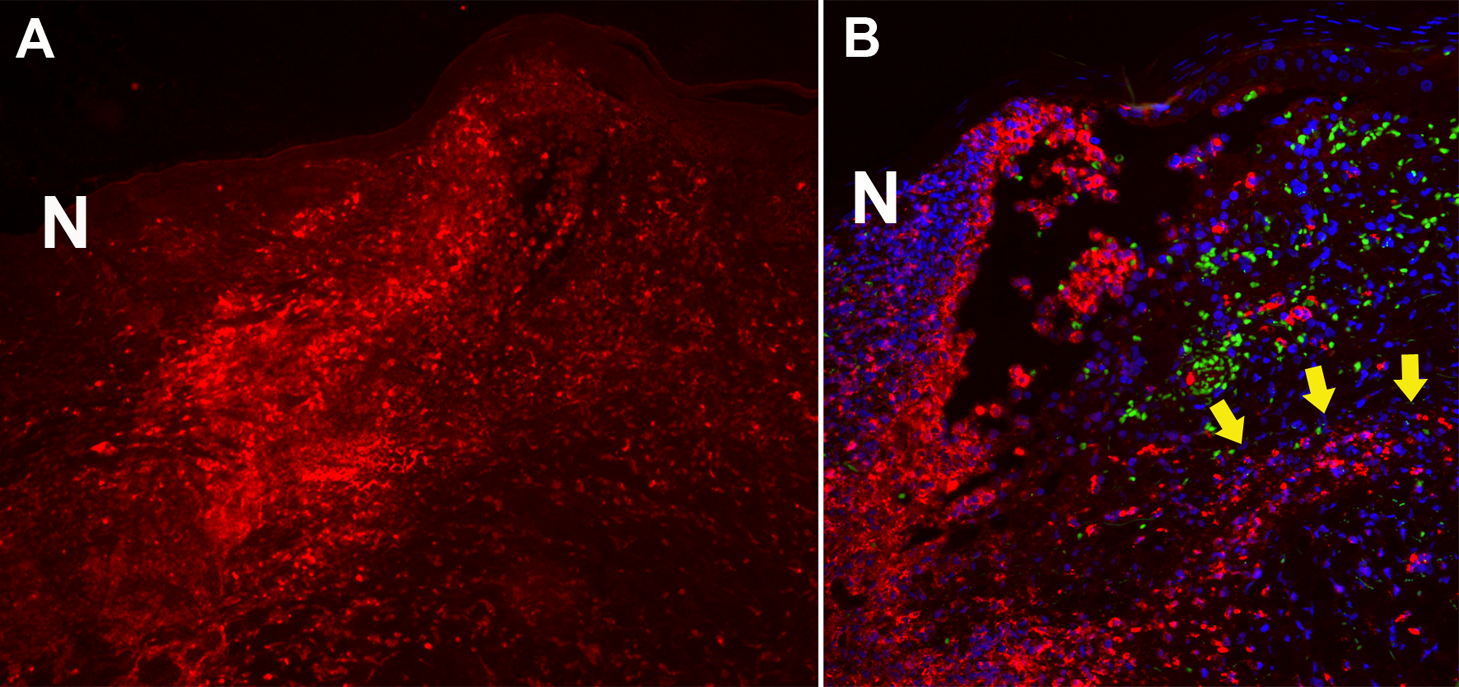

Supplement: Figure S2 — Neutrophil polymorphnuclear cells delineate the central necrotic zone of the eschar. The necrotic centre (labelled ‘N’, in upper left of both panels) is delineated by a granulocyte-dense zone depicted in red, buy immunofluorescent staining for neutrophil elastase NP57 (Panel A), and CD15 (Panel B). Accumulation of CD15+ neutrophils along the vasculature (yellow arrows) can be seen in panel B. Patient TM2193, magnification ×200, erythrocytes in yellow-green, NP57 and CD15 labeled in red, DAPI nuclear counterstain in blue. (TIFF) [file pntd.0001466.s002.tif]

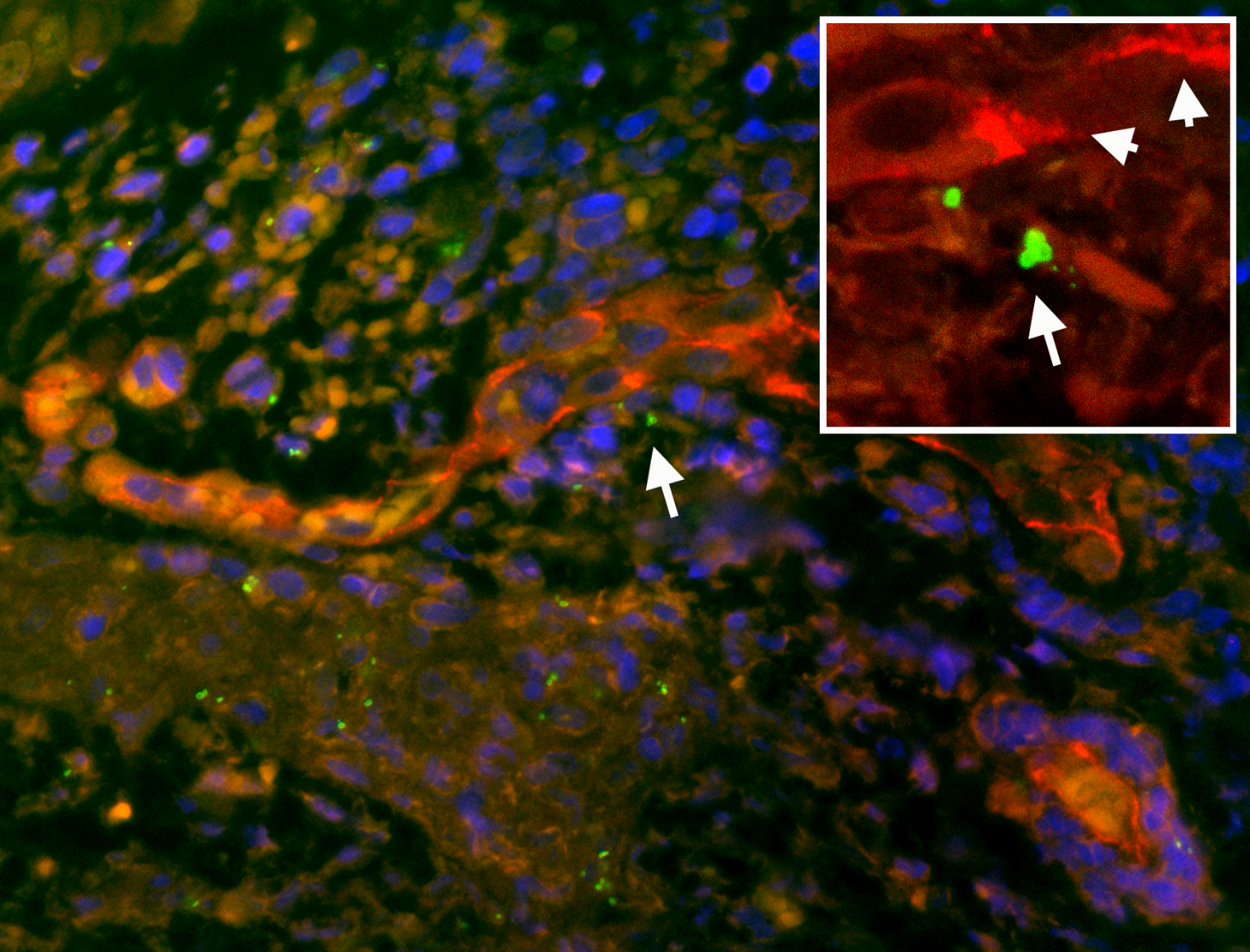

Supplement: Figure S3 — Longitudinal section of a superficial dermal blood vessel with perivascular mononuclear cells. A longitudinal cross-section of a blood vessel with endothelium stained by CD31 (in red) and O. tsutsugamushi (in green). A small cluster of O. tsutsugamushi (long white arrow) were examined using a LSM (insert). The insert is a 0.3 LSM micrograph with CD31+ cells (short arrows) with adjacent cells containing O. tsutsugamushi (long white arrow) One cell contains O. tsutsugamushi and three small ‘bleb-like’ inclusions, typically seen in HALDR-positive cells. Patient TM2193, magnification ×400, LSM insert ×1000, double-immunolabeling: CD31 in red, O. tsutsugamushi in green and DAPI nuclear counterstain in blue. (TIFF) [file pntd.0001466.s003.tif]
